# Gentiopicroside Ameliorates Diabetic Renal Tubulointerstitial Fibrosis *via* Inhibiting the AT1R/CK2/NF-κB Pathway

**DOI:** 10.3389/fphar.2022.848915

**Published:** 2022-06-23

**Authors:** Zhanchi Xu, Meng Zhang, Yu Wang, Rui Chen, Shiyue Xu, Xiaohong Sun, Yan Yang, Zeyuan Lin, Shaogui Wang, Heqing Huang

**Affiliations:** ^1^ School of Pharmaceutical Science, Guangzhou University of Chinese Medicine, Guangzhou, China; ^2^ School of Pharmaceutical Sciences, Sun Yat-sen University, Guangzhou, China; ^3^ The First Affiliated Hospital of Zhengzhou University, Zhengzhou,, China; ^4^ Department of Hypertension and Vascular Disease, The First Affiliated Hospital, Sun Yat-sen University, Guangzhou, China

**Keywords:** diabetic nephropathy, tubulointerstitial fibrosis, AT1R, CK2/NF-κB pathway, gentiopicroside

## Abstract

Renal tubulointerstitial fibrosis (TIF), characterized by epithelial-to-mesenchymal transition (EMT) of renal tubular epithelial cells, is the typical pathological alteration in diabetic nephropathy. Gentiopicroside (GPS), a natural compound with anti-inflammatory activity, has been demonstrated to alleviate glomerulosclerosis, whereas whether GPS inhibits TIF *via* regulating inflammation remains unclear. In this study, diabetic db/db mice and high glucose (HG)-stimulated renal tubular epithelial cells (NRK-52E) were applied to explore the effects and mechanisms of GPS on TIF. The results *in vivo* showed that GPS effectively improves glycolipid metabolism disorder, renal dysfunction, and TIF. In particular, GPS treatment reversed the abnormal expressions of EMT marker proteins including elevated α-smooth muscle actin and vimentin and decreased E-cadherin in the kidney of db/db mice. Moreover, GPS treatment also inhibited protein expressions of angiotensinⅡ type 1 receptor (AT1R) and CK2α and the activation of the NF-κB pathway. Importantly, the aforementioned effects of GPS acted *in vivo* were further observed *in vitro* in HG-stimulated NRK-52E cells, which were independent of its effects on glucose and lipid-lowering activity but were reversed by AT1R over-expression. Together, our results indicate that GPS that directly inhibits the CK2/NF-κB inflammatory signaling pathway *via* AT1R may also contribute to the amelioration of TIF in diabetes.

## Introduction

Diabetes is a global health problem with a high rate of morbidity and mortality (Standl et al., 2019). As the major microvascular complication of diabetes mellitus, diabetic nephropathy (DN) is the leading cause of diabetes-related death. However, the exact pathogenesis of DN is still unclear, and there is no targeted therapy that can effectively reverse the progress of DN (Danta et al., 2020). Therefore, it is urgent to find new therapeutic strategies and develop effective drugs for the treatment of DN.

Diabetic renal fibrosis is the main pathological change of DN, which is a combination of glomerulosclerosis and renal tubulointerstitial fibrosis (TIF) due to the excessive deposition of the extracellular matrix (ECM) (Boor et al., 2009; Adelusi et al., 2020). The tubulointerstitium accounts for more than 90% of renal parenchyma and performs a pivotal role in renal function. Recently, accumulating studies have shown that TIF is a key pathological factor of diabetic renal fibrosis and even the initiator of DN (Gilbert, 2017). It is acknowledged that epithelial-to-mesenchymal transition (EMT) is the main cause of TIF, which is characterized by the abnormal renal tubular epithelial cell marker proteins such as decreased E-cadherin (E-cad), increased α-smooth muscle actin (α-SMA), and vimentin (Tangrea et al., 2017). Importantly, EMT increases the migration and invasion of transformed epithelial cells, which causes excessive accumulation of ECM components such as fibronectin (FN) in the renal mesenchyme (Huang et al., 2019a; Huang et al., 2019b). Recently, it has been reported that inhibiting renal tubule EMT effectively attenuated DN, owing to the suppression of TIF (Yan et al., 2019). Therefore, inhibiting TIF may be a promising therapeutic strategy for DN.

The mechanism of TIF is complicated. It is believed that glycolipid metabolism disorder, non-enzymatic glycosylation of proteins, and oxidative stress are closely related to diabetic renal fibrosis including TIF (Reidy et al., 2014). Importantly, it has gradually become a consensus that chronic inflammation is the key factor of TIF, and activating NF-κB remarkably contributes to inflammation (Tornatore et al., 2012). IκBα, an endogenous inhibitory protein of NF-κB p65, anchors NF-κB p65 in the cytoplasm. However, IκBα is phosphorylated by kinase and degraded *via* the ubiquitin-proteasome pathway owing to various stimulations, which activate the NF-κB signaling pathway to trigger inflammation (Schmid et al., 2006). Our previous study showed that CK2α, the catalytic subunit of CK2, activates the NF-κB pathway by phosphorylating IκBα in DN (Huang et al., 2017). Therefore, the enhancement of IκBα *via* inhibiting CK2α considerably contributes to inhibiting NF-κB in diabetic TIF.

The angiotensinⅡ type 1 receptor (AT1R) is highly expressed in kidneys and facilitates the physiological functions induced by angiotensinⅡ (Hunyady and Catt, 2006). The most classical view of AT1R emphasizes its role as an endocrine regulator of renal sodium balance and blood pressure. But recent evidence pointed out its other pathological effects including inflammation and fibrosis (Ma et al., 2010). It is reported that AT1R promotes the phosphorylation of NF-κB at the p65 subunit, a key step in activating NF-κB, leading to the gene expression of chemokines interleukin-6 and inflammatory adhesion molecules in diabetes (Chao et al., 2017). Additionally, AT1R activation directly regulates the activity of many kinases. A recent study found that the activation of AT1R activated Src-family tyrosine kinase, thereby facilitating CK2 activation, which triggers the inflammation in cardiomyocytes (Kashihara et al., 2017). Therefore, it is promising that suppressing AT1R considerably contributes to the inhibition of the CK2/NF-κB signaling pathway, thereby attenuating inflammation in TIF.

Gentiopicroside (GPS) is a main active secoiridoid glycoside isolated from *Gentiana scabra* Bunge (Zhang et al., 2020). Intensive studies have demonstrated that GPS inhibited inflammation in various diseases (Lv et al., 2015). Additionally, our previous study indicated that GPS improved diabetic glomerular inflammatory fibrosis (Xiao et al., 2020). However, the effect of GPS on TIF in DN is still unclear. Based on the aforementioned analysis, we further investigated whether GPS inhibits the AT1R/CK2/NF-κB pathway to ameliorate diabetic TIF. The results of this study show that GPS effectively ameliorates diabetic TIF, and the mechanism is related to the inhibition of the AT1R/CK2/NF-κB pathway.

## Materials and Methods

### Reagents and Antibodies

D-glucose was obtained from AMRESCO (Ohio, United States). GPS used in animal experiments (purity>98%) was purchased from Nanjing Dilger Medical Technology Co., Ltd. (Nanjing, China). GPS used in *in vitro* experiment (purity>99%) was purchased from Nanjing ZheWeiKang Biological Technology Co., Ltd. (Nanjing, China). Dulbecco’s modified Eagle’s medium (DMEM) and fetal bovine serum (FBS) were purchased from Life Technologies (New York, United States). Furthermore, 10% goat serum was purchased from Boster Biological Technology Co., Ltd. (Wuhan, China). BCA Protein Assay Kit and ECL detection kit were purchased from Pierce (Illinois, United States). The secondary antibody used in immunofluorescence was purchased from Thermo (Massachusetts, United States). DAPI was purchased from the Beyotime Institute of Biotechnology (Shanghai, China). Secondary antibodies labeled with HRP were purchased from Promega (Wisconsin, United States). Polyvinylidene difluoride membrane was purchased from Millipore (Massachusetts, United States).

Antibodies against α-tubulin, FN, IκBα, phosphorylated IκBα (p-IκBα), and lamin B1 were purchased from Proteintech (Wuhan, China). Antibodies against p65 and CK2α were obtained from Santa Cruz (California, United States). Antibodies against α-SMA and E-cad were purchased from Cell Signaling Technology (Boston, United States). Antibody against vimentin was purchased from Boster Biological Technology Co., Ltd. (Wuhan, China). Antibody against ATR1 was purchased from Abcam (Massachusetts, United States). Antibody against phosphorylated CK2α (p-CK2α) was purchased from Affinity Biosciences (Ohio, United States). GFP antibody was purchased from the Beyotime Institute of Biotechnology (Shanghai, China).

### Animal Experiments

Male db/db mice (6 weeks of age) were randomly divided into the diabetic model group, low-dose GPS treatment group (50 mg/kg), medium-dose GPS treatment group (100 mg/kg), high-dose GPS treatment group (200 mg/kg), and valsartan (Val) treatment group (10 mg/kg) based on the fasting blood glucose (FBG) value. FBG was measured by using a glucometer (Johnson, New Jersey, United States) weekly. The number of animals in each group was 8. GPS and Val were intragastrically administered for 10 weeks. The mice in both the control group and diabetic model group were given the same volume of normal saline by intragastric administration. At the end of the experiment, 24 h urine was collected. After being sacrificed, the serum samples were separated and stored at −20°C for biochemical analysis. Kidney samples were fixed in 4% paraformaldehyde or frozen at −80°C. The immunohistochemical staining experiment was conducted as the reported method in Sawai et al. (2006).

Healthy male C57/BL6 mice (6 weeks of age) were used as the control group. Male C57/BL6 mice (Animal Quality Certificate Number: 44008500019074) were obtained from the experimental animal center of Sun Yat-sen University (Guangzhou, China). Male db/db mice (Animal Quality Certificate Number: 201919681) were purchased from GemPharmatech Co., Ltd. (Nanjing, China). Animals were housed under specific pathogen-free conditions. Experiments were implemented in accord with China Animal Welfare Legislation and were approved by the Ethics Committee on the Care and Use of Laboratory Animals of Sun Yat-sen University (Guangzhou, China).

### Biochemical Analysis

The kits used for biochemical analysis were provided by the Jiancheng Bioengineering Institute (Nanjing, China). The catalogs of serum creatinine (Src), blood urea nitrogen (BUN), and urine protein (Up) are C011-2-1, C013-2-1, and C035-2-1, respectively. The catalogs of glycated serum protein (GSP), hemoglobin A1c (HbA1c), triglycerides (TG), and low-density lipoprotein cholesterol (LDL-C) are A037-2, A056-1-1, A110-1-1, and A113-1-1, respectively. The aforementioned parameters were measured according to instructions.

### Cell Culture

NRK-52E cells were incubated in DMEM with 10% FBS at 37°C under an atmosphere of 5% CO_2_. When NRK-52E cells reached about 50% confluence, cells were cultured in a serum-free medium for 12–16 h and then divided into different groups. Subsequently, cells were treated with GPS for a specified time. In this study, the control group was cultured with 5.6 mM glucose, while the model group and GPS treatment group were cultured with 30 mM high glucose (HG).

### MTT assay

MTT assays were performed as the reported method in Xiao et al. (2020). The NRK-52E cells were treated with GPS for 36 h. A microplate reader (Omega Bio-Tek, Georgia, United States) was used for the determination of optical density at 570 nm.

### Cell Scratch Test

To investigate the migration ability of NRK-52E cells, cell scratch tests were performed as the reported method in Pang et al. (2020).

### Western Blot

The Western blot assay was conducted as the reported method in Xiao et al. (2020). Protein samples were extracted with RIPA buffer (Beyotime Institute of Biotechnology, Shanghai, China) containing protease and phosphatase inhibitors (Selleck Chemicals, Texas, United States). Nuclear and cytoplasmic proteins were extracted, according to the instruction of the protein extraction kit (Active Motif, Carlsbad, United States). The Tanon 5200 Chemiluminescent Imaging System (Shanghai Tanon Technology Co., Ltd., Shanghai, China) was applied to observe the target proteins. ImageJ software was used for quantitative analysis.

### Immunofluorescence

The immunofluorescence assay was conducted as the reported method in Xiao et al. (2020). A Zeiss LSM 510 laser confocal fluorescence microscope (Oberkochen, Germany) was used to collect images.

### Immunoprecipitation Assay

NRK-52E cells were lysed with immunoprecipitation (IP) lysate buffer (Beyotime Institute of Biotechnology, Shanghai, China) containing the protease inhibitor (Selleck Chemicals, Texas, United States). After that, the supernatants were collected. Next, the aforementioned cell lysates were incubated with 20 μL protein agarose A/G beads (Thermo, Massachusetts, United States), and the supernatants were obtained by transient centrifugation. Later, an antibody (against CK2α or IgG) was added with shaking for 24 h at 4°C. Subsequently, the aforementioned solutions were incubated with 20 μL protein agarose A/G beads for 4 h at 4°C. Beads were collected by transient centrifugation and washed with IP buffer 3 times. Finally, about 20 μL loading buffer (Fdbio, Hangzhou, China) was mixed with the beads and boiled for 5 min. The method of the Western blot was adopted to detect protein levels.

### Electrophoretic Mobility Shift Assay

The DNA-binding activity of transcription factor NF-κB was measured by electrophoretic mobility shift assay (EMSA). After that, the procedure was conducted according to the instruction of the Light Shift Chemiluminescent EMSA Kit (Pierce, Illinois, United States). The sequence of the biotin-labeled oligonucleotide probes for NF-κB (Beyotime Institute of Biotechnology, Shanghai, China) was as follows:

Sense: 5′-ACT​GAG​GGT​GAC​TCA​GCA​AAA​TC-3′
Antisense: 3′-TGA​CTC​CCA​CTG​AGT​CGT​TTT​AG-5′

The images were captured by using a Tanon 5200 Chemiluminescent Imaging System (Shanghai Tanon Technology Co., Ltd., Shanghai, China).

### Plasmid Transient Transfection

The pcDNA3.1-GFP-AT1R plasmid was obtained from GenePharma (Shanghai, China).

The plasmid was transfected to NRK-52E cells, according to the instruction of LTX & PLUS (Life Technologies, New York, United States). Briefly, NRK-52E cells were cultured in plates for 24 h prior to transfection, and then, 2 μg plasmids were transfected into cells by using LTX & PLUS. After 36 h of incubation, the medium was replaced with a serum-free medium for another 12 h. Then, the cells were harvested after further treatment.

### Dual Luciferase Reporter Assay

NRK-52E cells were cultured on 96-well plates with various interventions, according to specific experimental requirements. After that, the cells were co-transfected with 0.2 µg pNF-κB-Luc (Beyotime Institute of Biotechnology, Shanghai, China) and 0.04 µg pRL-TK (Promega, Wisconsin, United States). Next, collecting the cells and a dual-luciferase reporter assay system kit (Promega, Wisconsin, United States) was adopted to detect the luciferase activity. Renilla luciferase activity was used as an internal reference in this assay.

### Statistical Analysis

All experimental data were analyzed by the software GraphPad Prism 5.0. Values were expressed as mean ± standard deviation (SD). The data between the two groups were analyzed by the unpaired Student’s t-test. As for multiple comparisons, one-way ANOVA with *post hoc* multiple comparisons was adopted. A value of *p* < 0.05 was regarded as statistically significant. Independent experiments were performed at least three times with similar results.

## Results

### GPS Ameliorated Lipid and Glucose Metabolism Disorders in db/db Mice

**FIGURE 1:**
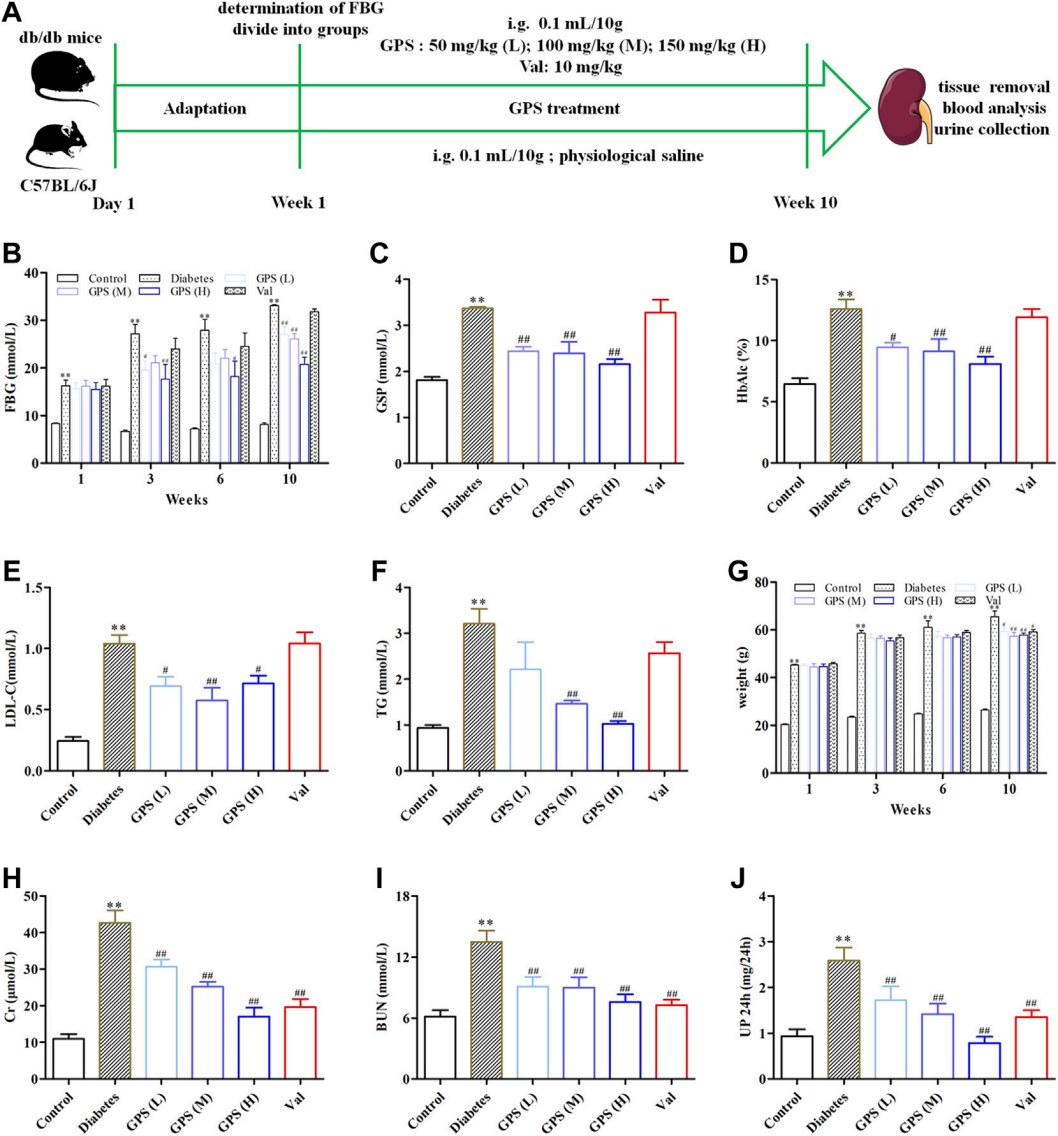
Effects of GPS on blood and urine biochemical parameters of diabetic mice. **(A)** Process of the experiment *in vivo*. **(B–D)** Levels of FBG, GSP, and HbAlc of experimental animals. **(E,F)** Levels of LDL-C and TG of experimental animals. **(G)** Body weight of experimental animals. **(H–J)** Levels of Cr, BUN, and 24 h Up of experimental animals. Data were expressed as means ± SD. *n* = 8. Diabetes: diabetes group; GPS (L): GPS treatment group (low dose: 50 mg/kg); GPS (M): GPS treatment group (medium dose: 100 mg/kg); GPS **(H)**: GPS treatment group (high dose: 200 mg/kg); Val: valsartan treatment group (10 mg/kg). **p < 0.01 *vs*. Ctrl. ^##^ *p* < 0.01 and ^#^ *p* < 0.05 *vs*. diabetes.

Hyperglycemia and dyslipidemia are closely related to the progress of diabetic renal fibrosis (Zoja et al., 2020). Therefore, the biochemical parameters related to glucose and lipid metabolism of db/db mice were determined after 10 weeks of GPS administration (Figure 1A). Compared to the control group, the FBG level was significantly increased in the diabetes group, which was effectively improved by GPS treatment (Figure 1B). Furthermore, GSP and HbA1c, other biomarkers for the assessment of long-term glycemic control, were also decreased after 10 weeks of GPS treatment (Figures 1C,D). In addition, GPS treatment notably decreased LDL-C and TG levels (Figures 1E,F). In addition to that, GPS reduced the body weight of db/db mice (Figure 1G). These results revealed GPS ameliorated lipid and glucose metabolism disorders in diabetic mice.

### GPS Ameliorated Renal Function and Inhibited Renal Tubulointerstitial Fibrosis in db/db Mice

Creatinine is the metabolite of muscle, and its amount is proportional to muscle mass. Physically, creatinine is cleared away from plasma by glomerular filtration and then excreted into the urine without tubular reabsorption. Therefore, the amount of Scr is constant under normal conditions, and an increased Scr level usually indicates the impaired renal function (Li et al., 2020). In addition to that, urea nitrogen, the main end product of protein metabolism, is another important parameter to evaluate the renal function. The elevated plasma urea nitrogen level is also a common clinical manifestation of DN (Wang et al., 2020). In addition, microalbuminuria is the early clinical sign of renal disease. Screening for microalbuminuria is crucially important for the diagnosis and management of DN (Kaburagi et al., 2019).

We assessed the effects of GPS on renal function in this assay. The results showed that the parameters related to renal function including Scr, BUN, and 24 Up were significantly increased in the model group. After the treatment of GPS, the levels of Scr, BUN, and 24 Up were remarkably reduced (Figures 1H–J). Thus, we concluded that GPS improved the renal function of db/db mice.

**FIGURE 2:**
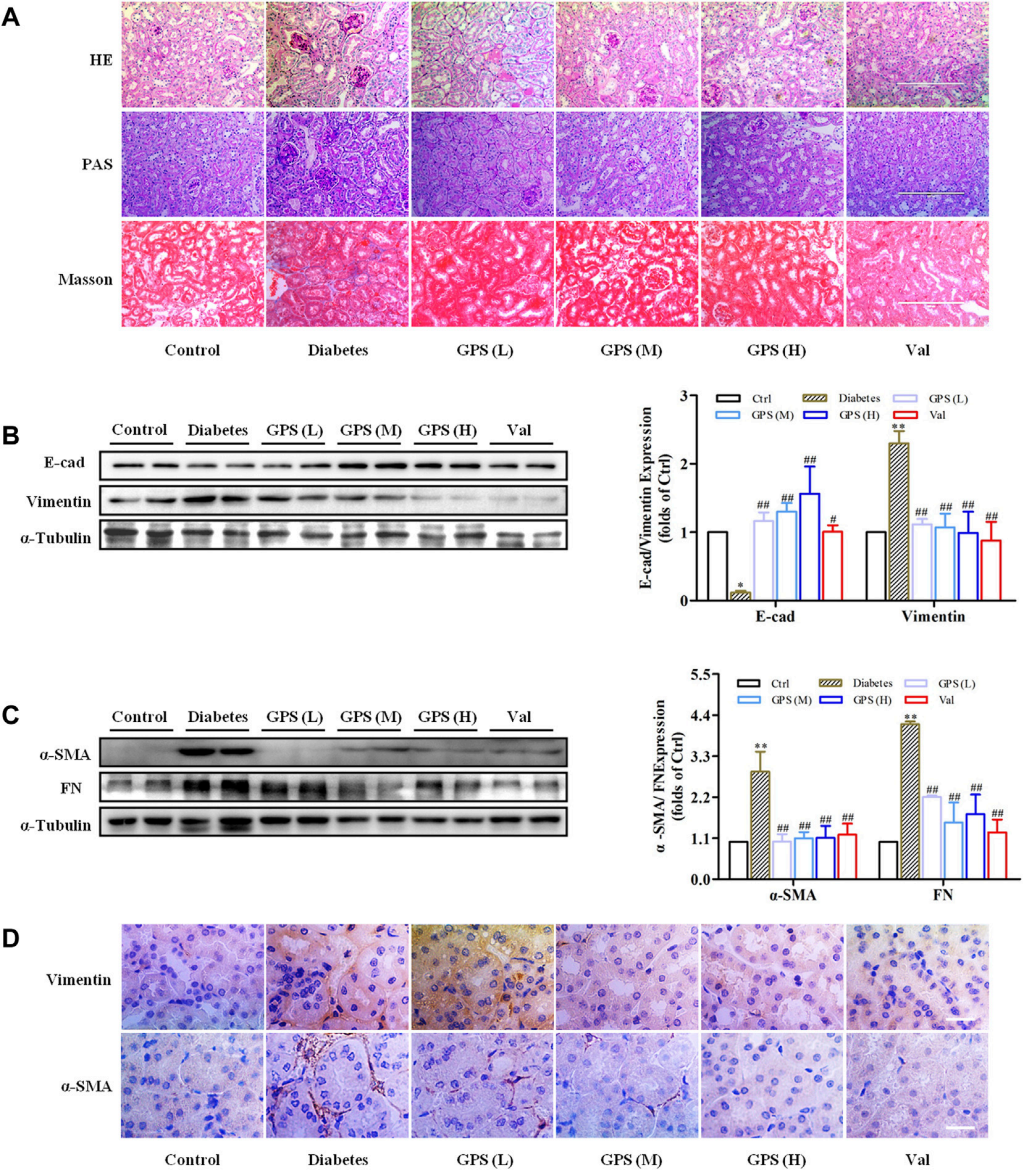
GPS treatment ameliorated renal fibrosis of diabetic mice. **(A)** Histopathology analysis of renal tubules by HE (scale bar: 200 μm), PAS (scale bar: 200 μm), and Masson (scale bar: 200 μm) staining. **(B,C)** Protein levels of E-cad, vimentin, α-SMA, and FN in renal tissues of diabetic mice were detected by Western blot. **(D)** Protein levels of vimentin and α-SMA in renal tubules of diabetic mice were detected by immunohistochemistry. Scale bar: 25 μm. Data were expressed as mean ± SD. *n* = 8. **p < 0.01 *vs*. Ctrl. ^##^ *p* < 0.01 and ^#^ *p* < 0.05 *vs*. diabetes.

Additionally, the methods of hematoxylin-eosin (HE), periodic acid–Schiff (PAS), and Masson staining were applied to investigate the effects of GPS on TIF. Lumen expansion was observed in the renal tubular of db/db mice by HE staining. PAS staining results showed that glycogen deposition in the renal cortex of db/db mice was increased when compared with the control group. Masson staining was used to detect the extent of collagen accumulation. The positively stained area in the renal tubular of db/db mice was significantly increased, indicating an increase in collagen accumulation in renal tissue. However, after GPS intervention, the lumen expansion gradually converted to normal, and the degrees of inflammation and fibrosis in the renal tubule were ameliorated (Figure 2A). Considering the vital role of renal tubular EMT in TIF, we further detected the effects of GPS on EMT marker proteins in kidney tissues of diabetic mice. Western blot results showed that the EMT biomarkers including FN, α-SMA, and vimentin were significantly decreased after GPS treatment, while E-cadherin protein expression was increased (Figures 2B–D, Supplementary Figures S1A,B). Taken together, the aforementioned results indicated that GPS ameliorated TIF in db/db mice.

### GPS Reduced the AT1R Protein Expression and Inhibited the CK2/NF-κB Pathway in db/db Mouse Kidneys

As mentioned earlier, highly activated AT1R may promote diabetic TIF by activating the CK2/NF-κB signaling pathway. Therefore, we speculated whether GPS suppressed the AT1R/CK2/NF-κB pathway.

**FIGURE 3:**
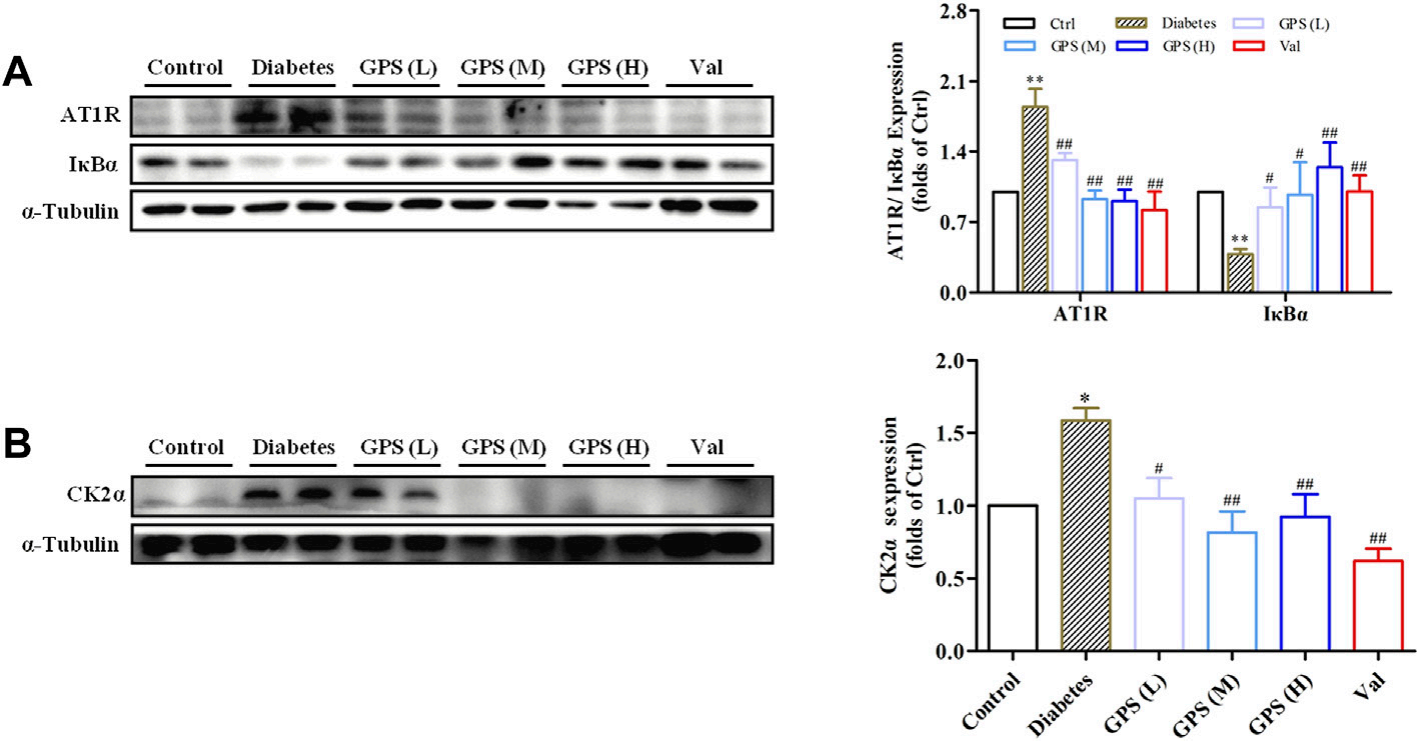
GPS inhibited the AT1R/CK2/NF-κB pathway in the kidney tissues of diabetic mice. **(A,B)** Protein levels of AT1R, IκBα, and CK2α in renal tissues of diabetic mice were detected by Western blot. Data were expressed as mean ± SD. *n* = 8. **p < 0.01 *vs*. Ctrl. ^##^ *p* < 0.01 and ^#^ *p* < 0.05 *vs*. diabetes.

First, the network pharmacology model was constructed. The result showed that interfering with the renin angiotensin system (RAS) was related to the treatment of DN with GPS (Supplementary Table S1). Next, we detected the influence of GPS on the AT1R/CK2/NF-κB pathway. The results showed that GPS treatment downregulated expressions of AT1R and CK2α in the kidney of db/db mice. Additionally, IκBα, the main endogenous inhibitory protein of NF-κB, was significantly upregulated by GPS treatment in kidney tissues of db/db mice, which indicates GPS could inhibit the activation of the NF-κB signaling pathway in kidneys of diabetic mice (Figures 3A,B). In summary, these results indicated that GPS inhibited the AT1R/CK2/NF-κB pathway in the renal tissue of db/db mice.

### GPS Inhibited the HG-Induced EMT Process in NRK-52E Cells

The results *in vivo* showed that GPS improved TIF and inhibited the AT1R/CK2/NF-κB pathway in the kidney of db/db mice. Chronic hyperglycemia is the key factor leading to TIF. In addition, growing evidence demonstrated that hyperglycemia was a vital contributor to the over-activation of RAS including AT1R in DN (Amorim et al., 2019). The results *in vivo* also showed that GPS markedly improved glycolipid metabolism disorder. Such a remarkable effect of GPS on glycolipid metabolism disorder in db/db mice may mainly contribute to the inhibition of TIF and the AT1R/CK2/NF-κB pathway. Therefore, the effects of GPS on TIF and the AT1R/CK2/NF-κB pathway in db/db mice may mainly be related to glucose lowering. However, whether GPS affects renal function directly was unclear. To further confirm the effects of GPS on TIF, renal tubular epithelial cell NRK-52E was applied to *in vitro* experiments.

**FIGURE 4:**
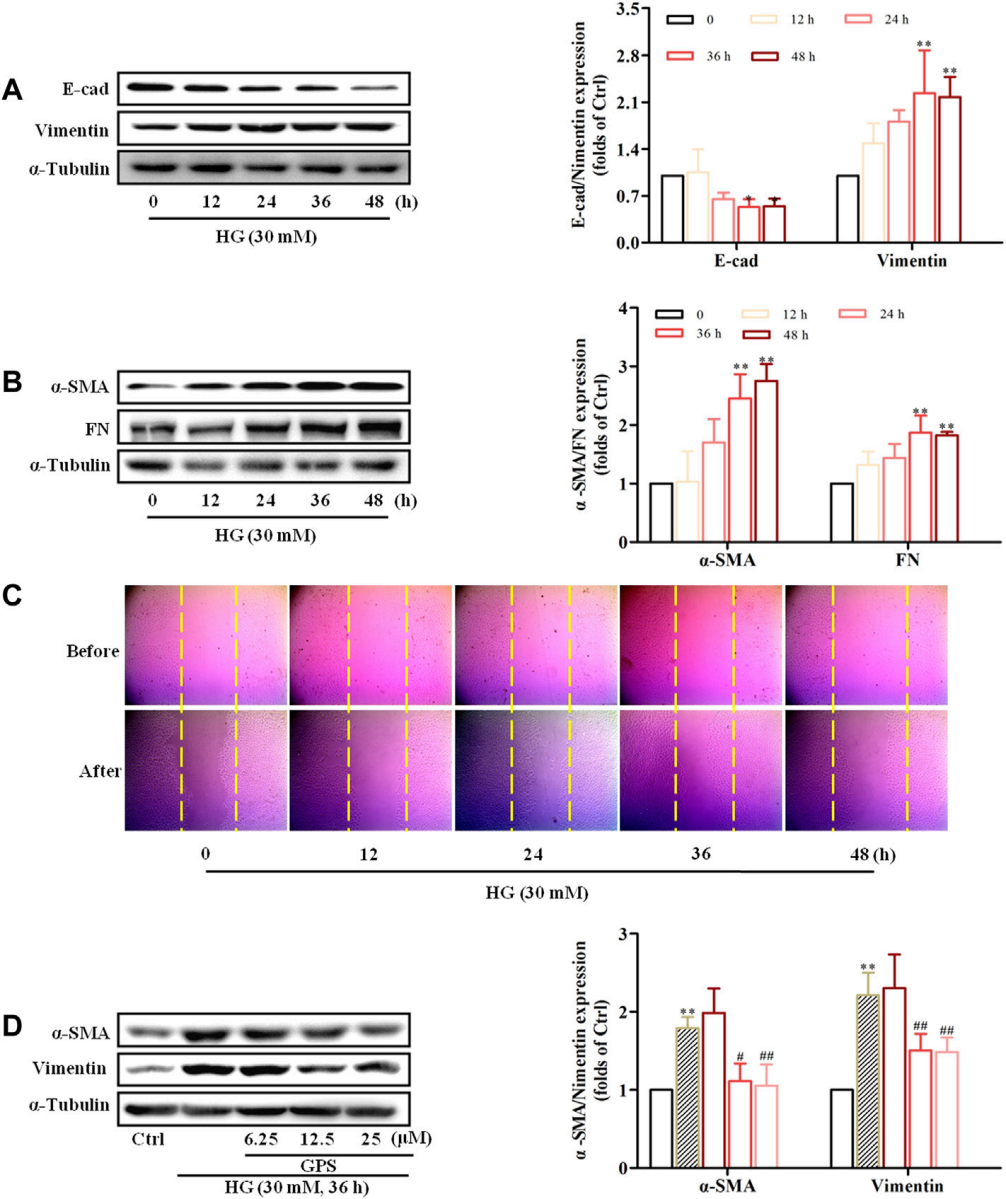
HG-induced EMT in NRK-52E cells and GPS inhibited HG-induced EMT in NRK-52E cells starting at 12.5 μM. (**A,B**) Protein levels of E-cad, vimentin, α-SMA, and FN under the HG conditions at various times were detected by Western blot. **p < 0.01 and *p < 0.05 *vs*. 0 h. **(C)** Migration ability of NRK-52E cells under the HG conditions at various times was detected by a cell scratch test. **p < 0.01 and *p < 0.05 *vs*. 0 h. **(D)** Protein levels of α-SMA and vimentin after GPS intervention were detected by Western blot. **p < 0.01 *vs*. Ctrl, ^##^ *p* < 0.01 *vs*. HG. Data were expressed as mean ± SD.

Western blot results showed that E-cad expression was decreased, and the protein expressions of α-SMA, vimentin, and FN were increased in NRK-52E cells stimulated with HG for 36 h (Figures 4A,B). Additionally, the migration ability of NRK-52E cells was significantly increased after HG treatment for 36 h (Figure 4C, Supplementary Figure S1C). Therefore, the treatment of HG for 36 h was adopted for the forthcoming studies.

MTT results showed that GPS with concentrations less than 800 μM had no cytotoxicity to NRK-52E cells (Supplementary Figure S1D). Moreover, Western blot results showed that GPS suppressed the expressions of vimentin and α-SMA starting at 12.5 μM in HG-treated NRK-52E cells (Figure 4D). Therefore, 12.5, 25, and 50 μM GPS were adopted in subsequent experiments.

**FIGURE 5:**
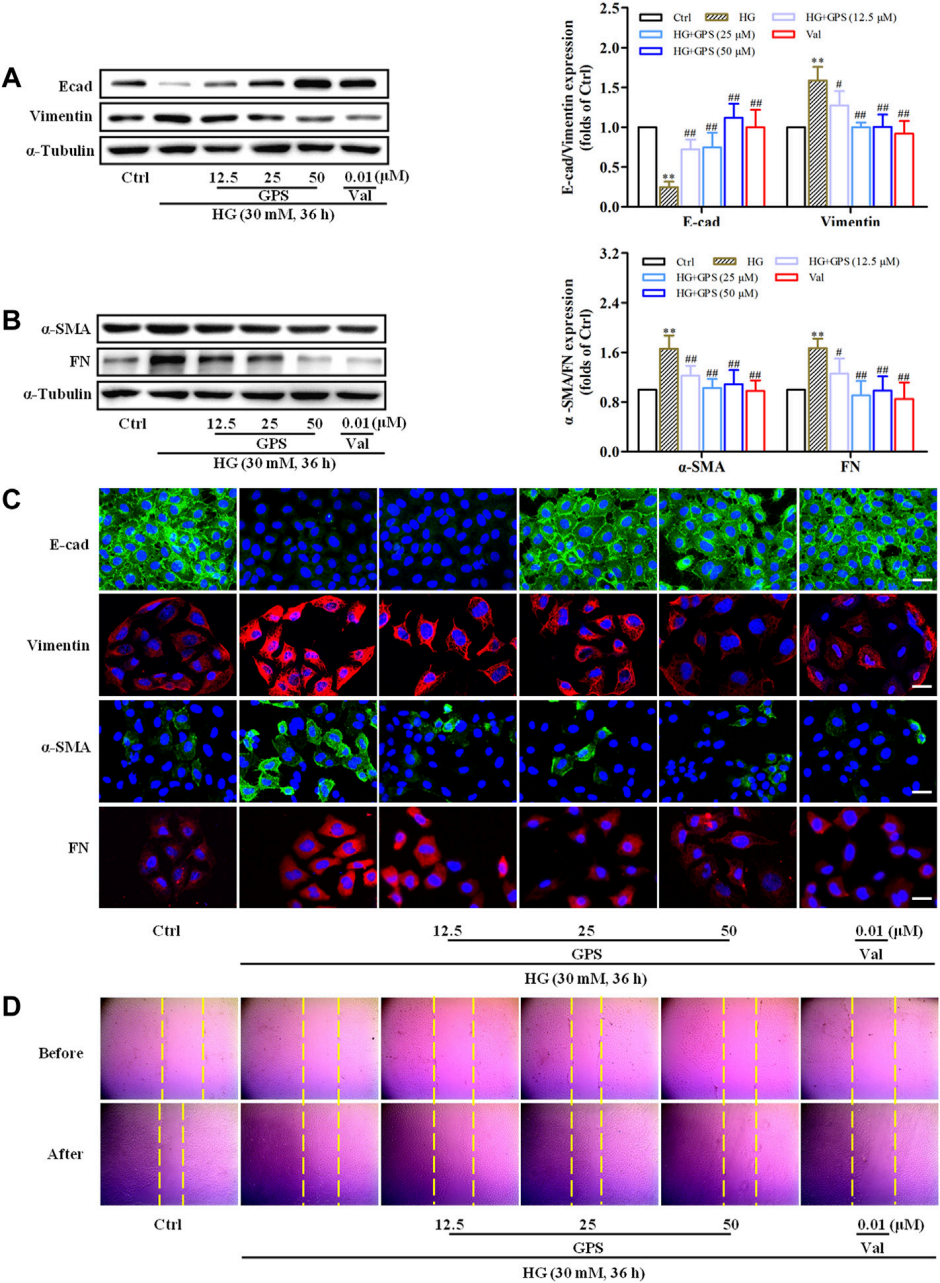
GPS inhibited HG-induced EMT in NRK-52E cells. **(A,B)** Protein levels of E-cad, vimentin, α-SMA, and FN after GPS intervention were detected by Western blot. **(C)** Protein levels of E-cad, vimentin, α-SMA, and FN after GPS intervention were detected by immunofluorescence. Scale bar: 20 μm. **(D)** Migration ability of NRK-52E cells after GPS intervention was detected by a cell scratch test. **p < 0.01 *vs*. Ctrl, ^##^ *p* < 0.01 and ^#^ *p* < 0.05 *vs*. HG. Data were expressed as mean ± SD.

GPS reduced expressions of FN, vimentin, and α-SMA and promoted the E-cad expression in HG-induced NRK-52E cells (Figures 5A–C). Moreover, the migration ability was decreased after GPS treatment (Figure 5D, Supplementary Figure S1E). The aforementioned results indicated that GPS inhibited the HG-induced EMT process in NRK-52E cells.

### GPS Inhibited HG-Induced Activation of the AT1R/CK2/NF-κB Pathway in NRK-52E Cells

After HG stimulation for 30 min, the NF-κB p65 expression in the nucleus was significantly increased (Supplementary Figure S1F). Thus, the effect of GPS on the CK2/NF-κB pathway was studied after HG stimulation for 30 min.

**FIGURE 6:**
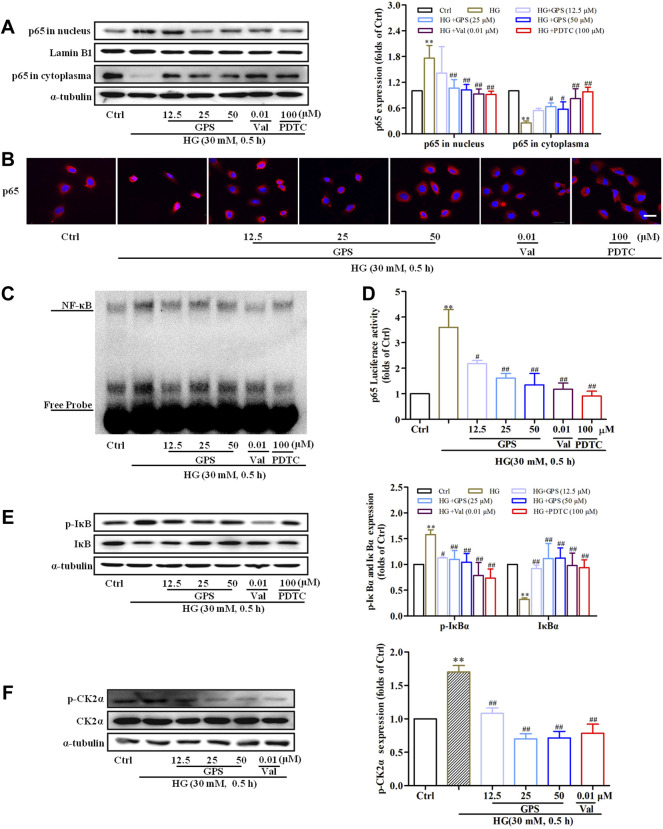
GPS inhibited HG-induced activation of the CK2/NF-κB pathway in NRK-52E cells. **(A)** Effects of GPS on p65 distribution in the nucleus and cytoplasm were detected by Western blot. **p < 0.01 *vs*. Ctrl. ^##^ *p* < 0.01 and ^#^ *p* < 0.05 *vs*. HG. **(B)** Effects of GPS on p65 distribution in the nucleus and cytoplasm were detected by immunofluorescence. Scale bar: 20 μm. **(C)** Effect of GPS on the NF-κB p65 DNA binding activity was detected by EMSA. **(D)** Effect of GPS on the NF-κB p65 transcriptional activity was detected by luciferase reporter assay. **p < 0.01 *vs*. Ctrl. ^##^ *p* < 0.01 and ^#^ *p* < 0.05 *vs*. HG. **(E,F)** Protein levels of IκBα, p-IκBα, and p-CK2α after GPS intervention were detected by Western blot. **p < 0.01 and *p < 0.05 *vs*. Ctrl. ^##^ *p* < 0.01 and ^#^ *p* < 0.05 *vs*. HG. **p < 0.01 *vs*. Ctrl. ^##^ *p* < 0.01 and ^#^ *p* < 0.05 *vs*. HG. Data were expressed as mean ± SD.

The result indicated that GPS effectively inhibited the nuclear expression of NF-κB p65, which was consistent with the effect of PDTC (NF-κB inhibitor) (Figures 6A,B). EMSA showed that HG stimulation increased the binding of NF-κB to target probes, but GPS intervention inhibited the aforementioned phenomena (Figure 6C). Furthermore, the dual luciferase reporter gene assay showed that the NF-κB p65 transcriptional activity was decreased after GPS treatment (Figure 6D).

Physiologically, IκBα, as the anchor protein of NF-κB p65, binds with NF-κB p65 in the cytoplasm. In response to HG stimulation, IκBα is phosphorylated by upstream kinase and then degraded after ubiquitination. Subsequently, NF-κB p65 translocates into the nucleus (Wu et al., 2020). Therefore, the levels of IκBα and phosphorylated IκB were measured in this assay. The decreased phosphorylation level and increased total protein expression of IκBα were observed after the intervention of GPS (Figure 6E). The phosphorylation of CK2α at the site of threonine 360 was an indicator of CK2α kinase activity (Ota et al., 2020). We further tested the effect of GPS on the phosphorylation of CK2α at the site of threonine 360. Western blot data showed that GPS significantly downregulated the p-CK2α expression as compared with HG-treated cells (Figure 6F).

**FIGURE 7:**
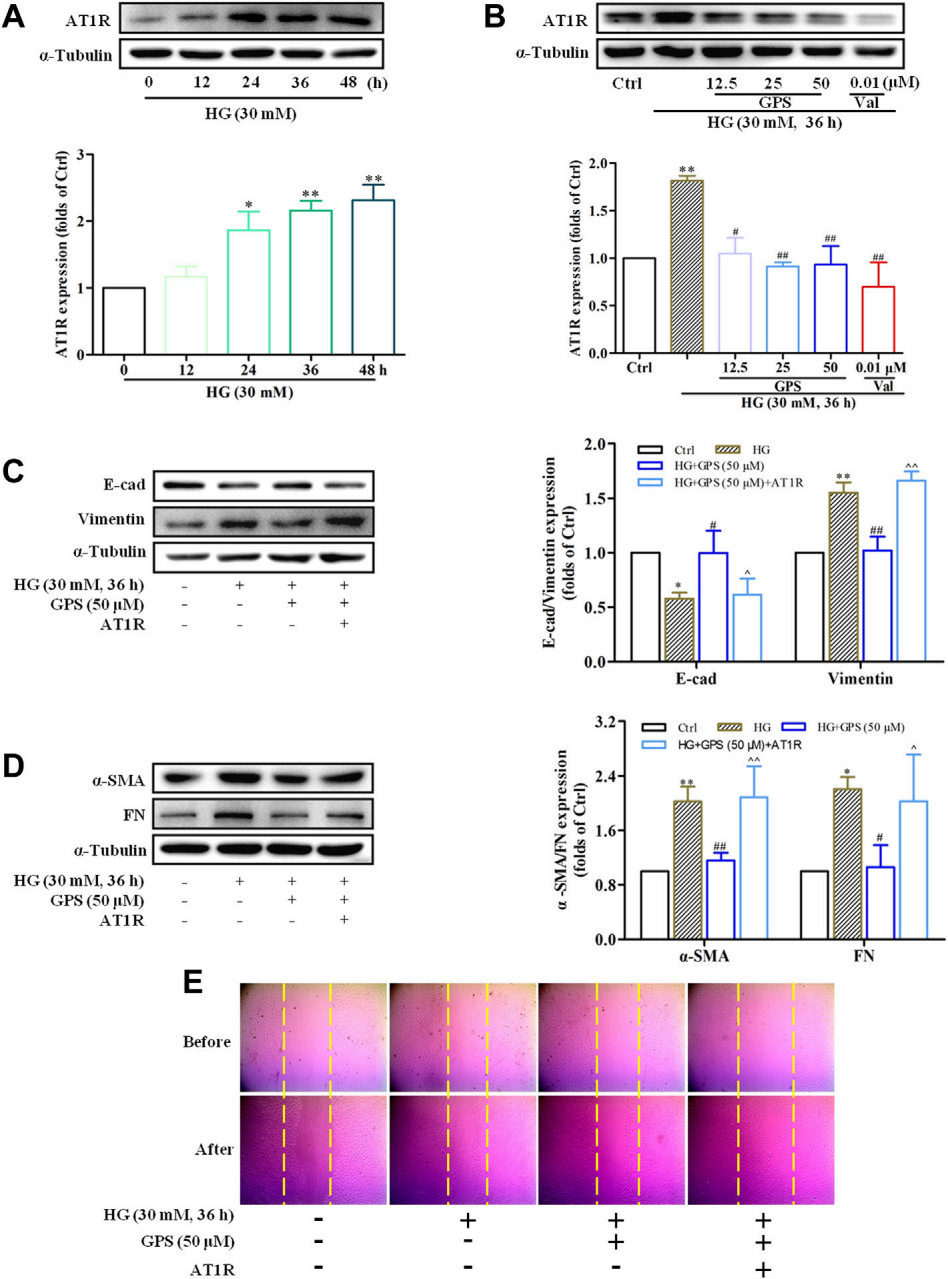
GPS downregulated the protein level of AT1R, and AT1R over-expression reversed the effect of GPS on EMT in HG-induced NRK-52E cells. **(A)** Expressions of AT1R under the HG conditions at various times were detected by Western blot. **p < 0.01 and *p < 0.05 *vs*. 0 h. **(B)** Protein level of AT1R after GPS intervention was detected by Western blot. **p < 0.01 *vs*. Ctrl. ^##^ *p* < 0.01 and ^#^ *p* < 0.05 *vs*. HG. **(C,D)** Effects of GPS on E-cad, vimentin, α-SMA, and FN expression after AT1R over-expression were detected by Western blot. **p < 0.01 *vs*. Ctrl. ^##^ *p* < 0.01 and ^#^ *p* < 0.05 *vs*. HG. ^^*p* < 0.05 *vs*. HG with GPS. **(E)** Effect of GPS on the migration ability after the AT1R over-expression was detected by the cell scratch test. Data were expressed as mean ± SD.

In addition to that, the effect of GPS on AT1R was explored in this assay. Western blot data showed that the AT1R expression was upregulated under the HG condition (Figure 7A), while GPS significantly downregulated the AT1R expression (Figure 7B). In summary, GPS inhibited HG-induced activation of the AT1R/CK2/NF-κB pathway in NRK-52E cells.

### AT1R Over-Expression Reversed the Inhibitory Effect of GPS on EMT

In order to further clarify the significance of AT1R in this process, NRK-52E cells were transfected with the exogenous AT1R plasmids, and corresponding validation experiments were conducted.

Western blot results showed that the AT1R expression was significantly increased accompanied by a striking increase in the GFP expression after the NRKA-52E cells were transfected with over-expression plasmid targeting AT1R (Supplementary Figure S2A). Compared with the GPS intervention group, the protein expressions of FN, vimentin, and α-SMA were increased, and E-cad expression was reduced when GPS and the over-expression plasmid of AT1R were given at the same time (Figures 7C,D). Moreover, the scratch test also showed that the inhibitory effect of GPS on the migration ability of NRK-52E cells was reversed by the AT1R over-expression (Figure 7E, Supplementary Figure S2B). Therefore, these results indicated that GPS inhibited the HG-induced EMT process through AT1R.

### AT1R Over-Expression Abolished the Inhibitory Effect of GPS on the CK2/NF-κB Pathway

**FIGURE 8:**
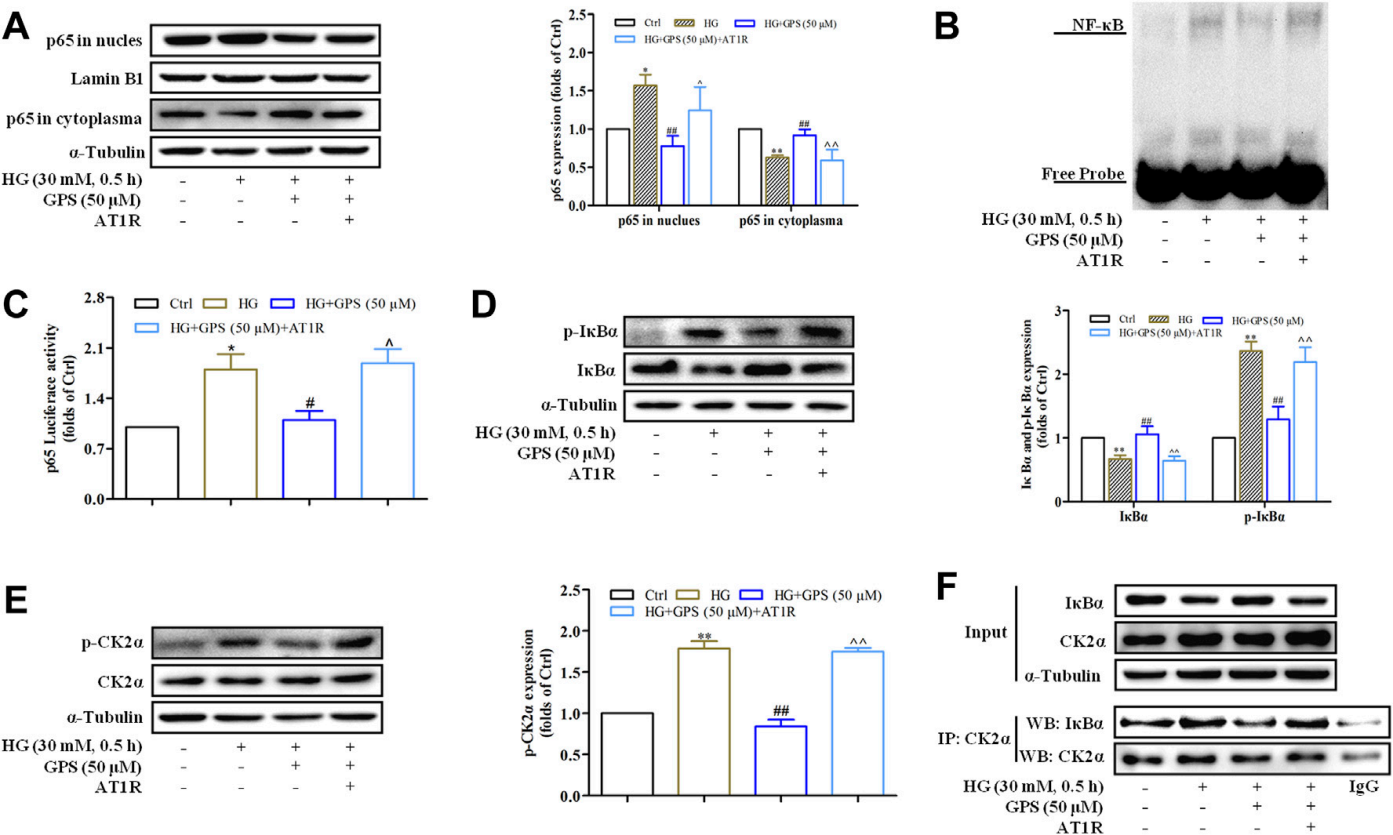
AT1R over-expression reversed the effect of GPS on regulating the CK2/NF-κB pathway. **(A)** Effects of GPS on p65 distribution in the nucleus and cytoplasm after AT1R over-expression were detected by Western blot. **(B)** Effects of GPS on the DNA NF-κB p65 binding activity after the AT1R over-expression were detected by EMSA. **(C)** Effect of GPS on the NF-κB p65 transcriptional activity after the AT1R over-expression was detected by luciferase reporter assay. **(D,E)** Effects of GPS on protein expressions of p-IκBα, IκBα, and p-CK2α after AT1R over-expression was detected by Western blot. **(F)** Binding of CK2α and IκBα in NRK-52E was detected by immunoprecipitation. **p < 0.01 and *p < 0.05 *vs*. Ctrl. ^##^ *p* < 0.01 and ^#^ *p* < 0.05 *vs*. HG. ^^*p* < 0.01 and ^*p* < 0.05 *vs*. HG with GPS. Data were expressed as mean ± SD.

Western blot results showed that the effects of GPS on NF-κB p65 nuclear translocation, NF-κB binding activation, and NF-κB transcriptional activity were abolished after the overexpression of AT1R (Figures 8A–C, Supplementary Figure S2C). In addition, the downregulatory effects of GPS on IκBα and p-CK2α levels were reversed by the overexpression of AT1R (Figures 8D,E). Furthermore, the IP assay showed that the interaction of CK2α with IκBα was increased in HG-induced NRK-52E cells. Importantly, GPS effectively inhibited the interaction of CK2α with IκBα in HG-induced cells. Notably, the downregulatory effect of GPS on the interaction was reversed after the overexpression of AT1R (Figure 8F, Supplementary Figure S2D).

Taken together, these observations further confirmed that inhibiting the CK2/NF-κB pathway *via* AT1R is one of the mechanisms for GPS to improve EMT in TIF.

## Discussion

Microalbuminuria is widely applied as a diagnostic index of DN, which is related to glomerular filtration barrier damage and glomerular ultrafiltration. However, a study showed that decreased albumin absorption and increased excretion were also observed in renal tubular epithelial cells of diabetic rats, but there was no significant change in the glomerular filtration rate (Tojo et al., 2001). Additionally, it was reported that the expression of Na-glucose cotransporter 2 in renal tubular epithelial cells was increased in DN. Subsequently, upregulated Na-glucose cotransporter 2 facilitates hyperfiltration by influencing the fluid and NaCl reabsorption through the proximal tubule, thereby inhibiting a tubuloglomerular feedback response (Chan et al., 2012). Therefore, these emphasize the crucial role of the renal tubule in DN, and the improvement of tubular injury is also of great importance for the treatment of DN. Our previous study indicated that GPS improved diabetic glomerular fibrosis *via* inhibition of inflammation in STZ-induced diabetic mice and HG-induced glomerular mesangial cells (Xiao et al., 2020). However, whether GPS improves diabetic TIF needs further exploration.

It is recognized that db/db mice are a spontaneous diabetic model that can generate the pathological characteristics of DN (Azushima et al., 2017). *In vivo* results have shown that the abnormal glycolipid indicators of db/db mice were improved by GPS. Importantly, GPS effectively improved diabetic TIF. Tubular epithelial cells, the most prominent cell type in the renal cortical tubular interstitium, could be transformed into a myofibroblast phenotype under diabetes conditions and eventually lead to abnormal ECM accumulation in the renal interstitium (Chen et al., 2018). Thus, EMT is recognized as a key contributor to TIF, and the inhibition of renal tubular EMT can prevent TIF. In the present study, GPS reversed the abnormal expressions of the EMT biomarker in the renal tissue of db/db mice.

DN, characterized by TIF, is closely related to an inflammatory response (Meng et al., 2014). Clinical studies have shown that inflammatory markers are significantly upregulated in DN patients (Mora and Navarro, 2004). NF-κB is the crucial signaling pathway mediating the inflammatory response. Activating the NF-κB pathway enhanced the inflammatory marker expressions such as transforming growth factor-β1, adhesion molecular, and monocyte chemokine, which are the potent inducers of renal tubular EMT (Nam et al., 2008; López-Novoa and Nieto, 2009). Therefore, inhibiting the NF-κB pathway considerably contributes to the prevention of EMT in TIF. Recently, the role of CK2 in DN has gained attention. Our previous study showed that CK2α was activated in AGE-induced glomerular mesangial cells and triggered the over-production of inflammatory adhesion molecules and ECM. Additionally, CK2α promoted the activation of NF-κB *via* phosphorylating IκBα in DN (Huang et al., 2017). It is gradually confirmed that AT1R causes kidney damage through a series of non-hemodynamic pathways such as pro-inflammation and pro-fibrosis (Cantero-Navarro et al., 2021). Moreover, AT1R positively regulated the activation of CK2α (Li et al., 2015). Therefore, inhibiting the AT1R/CK2/NF-κB pathway effectively prevents EMT in TIF. In the present study, we found that GPS not only prevented the CK2/NF-κB pathway but also inhibited the AT1R expression in the renal tissue of db/db mice. These results suggested that GPS might inhibit EMT in TIF *via* the AT1R/CK2/NF-κB pathway.

Chronic hyperglycemia is the key factor leading to DN. Classically, DN is initially caused by high intraglomerular pressure, which was driven by hyperglycemia. High intraglomerular pressure develops to the injury of the glomerular filtration barrier and ultimately renal fibrosis (Forbes et al., 2007; Márquez et al., 2015; Amorim et al., 2019). Moreover, hyperglycemia stimulates AngⅡ production which favors AT1R activation (Amorim et al., 2019). Over-activated AngⅡ/AT1R axis promotes progressive inflammatory injury in DN *via* activating the NF-κB pathway (Pandey et al., 2015). Therefore, the activation of the AT1R/CK2/NF-κB pathway in renal tissue and the development of EMT in TIF are influenced by glycemic control. Our previous study demonstrated that GPS reduced the blood glucose content in STZ-induced diabetic mice (Xiao et al., 2022). The present study also showed that the treatment with GPS largely reduced blood glucose levels in diabetic db/db mice, which may mainly contribute to the amelioration of EMT in TIF and the inhibition of the AT1R/CK2/NF-κB pathway in the kidney of db/db mice. Interestingly, GPS also directly prevented EMT by inhibiting the AT1R/CK2/NF-κB pathway in HG-induced renal tubular epithelial cells. Collectively, GPS inhibits TIF, which may be related to its pharmacological effect of glucose-lowering activity and direct inhibition of EMT.

AT1R blockers are the current standard of therapy to prevent proteinuric chronic kidney disease progression (Tamargo and Tamargo, 2017). Therefore, Val, an AT1R blocker, was chosen as the positive control in our study. Our present study indicated that GPS showed competent effects with Val in improving the renal function. Additionally, GPS ameliorated lipid and glucose metabolism disorders of diabetic mice, which showed certain superiority as compared with Val.

The number of diabetes patients in the world has reached 415 million and is expected to increase to 642 million by 2040 (Standl et al., 2019). DN is the most common microvascular complication of diabetes and is regarded as the leading cause of end-stage kidney disease. However, there is no targeted therapy that can effectively reverse the progress of DN. Developing effective drugs is of great significance. DN is the result of multiple factors. The treatment of DN requires both hypoglycemic and antifibrotic therapy. GPS, a natural product mainly isolated from *Gentiana scabra* Bunge may be a potential candidate drug for DN treatment, owing to its safety and multiple pharmacological effects against inflammation, fibrosis, hyperglycemia, and hyperlipidemia (Xiao et al., 2020). This study explores the effect and mechanism of GPS on TIF. Our study lays the foundation for the clinical application of GPS for DN treatment and the development of medicinal plant resources such as *Gentiana scabra* Bunge.

## Conclusion

GPS inhibits TIF, which may be related to its pharmacological effect of glucose-lowering activity. Moreover, GPS may also directly inhibit the CK2/NF-κB inflammatory signaling pathway *via* AT1R to ameliorate TIF in diabetes.

## Data Availability

The original contributions presented in the study are included in the article/Supplementary Material; further inquiries can be directed to the corresponding authors.

## Glossary

ACE: angiotensin-converting enzyme
AT1R: angiotensinⅡ type 1 receptor
BUN: blood urea nitrogen
DMEM: Dulbecco’s modified Eagle medium
DN: diabetic nephropathy
E-cad: E-cadherin
ECM: extracellular matrix
EMSA: electrophoretic mobility shift assay
EMT: epithelial-to-mesenchymal transition
FBG: fasting blood glucose
FBS: fetal bovine serum
FN: fibronectin
GPS: gentiopicroside
GSP: glycated serum protein
HbA1c: hemoglobin A1c
HE: hematoxylin-eosin
HG: high glucose
IP: immunoprecipitation
LDL-C: low-density lipoprotein cholesterol
PAS: periodic acid–Schiff
p-CK2α: phosphorylated CK2α
p-IκBα: phosphorylated IκBα
RAS: renin angiotensin system
SD: standard deviation
Src: serum creatinine
TG: triglycerides
TIF: tubulointerstitial fibrosis
Up: urine protein
Val: valsartan
α-SMA: α-smooth muscle actin
