# Supplementary material for: Gentiopicroside Ameliorates Diabetic Renal Tubulointerstitial Fibrosis via Inhibiting the AT1R/CK2/NF-κB Pathway
Source: Front Pharmacol. 2022 Jun 23;13:848915. doi: 10.3389/fphar.2022.848915 (PMC9260113; doi:10.3389/fphar.2022.848915)
Supplement: Supplementary file 1 [file DataSheet1.pdf]

Supplementary Material

Gentiopicroside ameliorates diabetic renal tubulointerstitial fibrosis via inhibiting AT1R/CK2/NF-κB pathway

Zhanchi Xu [1,2†], Meng Zhang [2†], Yu Wang [4†], Rui Chen [2], Shiyue Xu [3*], Xiaohong Sun [2], Yan Yang [2], Zeyuan Lin [2], Shaogui Wang [1*], Heqing Huang [2*]
[1] School of Pharmaceutical Science, Guangzhou University of Chinese Medicine, Guangzhou 510006, China
[2] School of Pharmaceutical Sciences, Sun Yat-sen University, Guangzhou 510006, China
[3] Department of Hypertension and Vascular Disease, The First Affiliated Hospital, Sun Yat-sen University, Guangzhou, 510080, China
[4] The First Affiliated Hospital of Zhengzhou University, Zhengzhou, 450052, China

*** Correspondence:**
Heqing Huang
huangheq@mail.sysu.edu.cn
Shaogui Wang
wangshaogui@gzucm.edu.cn
Shiyue Xu
xushy25@mail.sysu.edu.cn

[†]These authors have contributed equally to this work

1. The effects of GPS on Vimentin and α-SMA in db/db mice were detected by immunohistochemistry

The results of immunohistochemistry were quantitatively analyzed by the software of Image J. The data showed that GPS treatment could inhibit the high expressions of α-SMA and Vimentin in db/db mice (**Supplement Figures S1A,B**).

2. Mechanism of GPS for DN treatment was predicted based on network pharmacology

The method of network pharmacology was adopted to predict the mechanism of GPS for DN treatment according to reported method (Xiong et al., 2020**)**. Firstly, the chemical structure and 3D file of the GPS were obtained from PubChem (https://pubchem.ncbi.nlm.nih.gov/). Then potential targets of GPS were obtained from the Swiss Target Prediction (http: //www.swisstargetprediction.ch). After that, some target manes need to be normalized by Uniprot (https://www.uniprot.org/) and Homo sapiens were kept for further analysis. The search keyword "diabetic nephropathy" was used in Gene Cards database (https://www.genecards.org/) and DN related genes were obtained. Next, the DN-related genes were compared with the potential targets of GPS, providing potential targets of GPS for anti-DN. Later, above targets were entered into String database (https: //string-db.org/) with the species being limited to Homo sapiens, and the Protein-Protein Interaction (PPI) network with a score greater than 0.7 was retained. The PPI was obtained and the results of the Kyoto Encyclopedia of Genesand Genomes (KEGG) pathway annotation were used to analyze the PPI network and the key proteins were selected for further analysis.From above analysis, we known that intervening RAS was the main mechanism of GPS for DN treatment (**Supplement Table 1**).

3. HG increased migration ability of NRK-52E cells

The effect of HG on the migration ability of NRK-52E cells was detected by cell scratch test, and scratch areas were quantitatively analyzed by the software of Image J (**Supplement Figure S1C**).

4. ***MTT assay***

MTT assay showed that GPS with the concentration less than 800 μM had no cytotoxicity to NRK-52E cells (**Supplement Figure S1D**).

5. GPS inhibited HG-induced migration of NRK-52E cells

The effect of GPS on the migration ability of HG-induced NRK-52E cells was detected by cell scratch test, and scratch areas were quantitatively analyzed by the software of Image J (**Supplement Figure S1E**).

6. The distributions of p65 in nucleus and cytoplasm under the HG conditions at various times were detected by western blot

After stimulation with HG for 30 min, the p65 expression in nucleus was significantly increased (**Supplement Figure S1F**).

7. Plasmid transient transfection

Compared with normal group, AT1R expression significantly increased accompanied by a striking increase in GFP expression after the NRKA-52E cells were transfected with over expression plasmid targeting AT1R (**Supplement Figure S2A**).

8. AT1R over-expression can reverse the inhibitory effect of GPS on NRK-52E cells migration

The scratch areas were quantified by the software of Image J. The data showed that the migration ability of NRK-52E cells incubated with HG was increased obviously and the decreased migration was observed after cells were treated with GPS. However, the increased migration was detected again after cells were treated with GPS and over expression plasmid of AT1R (**Supplement Figure S2B**).

9. AT1R over-expression can reverse the inhibitory effect of GPS on p65 distributed in nucleus

As shown in immunofluorescence, the phenomenon of p65 translocation into nucleus was inhibited after GPS intervention. Nevertheless, AT1R over-expression up-regulated the expression of p65 in nucleus (**Supplement Figure S2C**).

10. The co-localization of CK2α and IκBα in cytoplasm

As shown in immunofluorescence, the co-localization of CK2α and IκBα in cytoplasm was observed in NRK-52E cells (**Supplement Figure S2D**).

References:

Xiong, W.-c., Wu, H.-z., Xiong, Y.-y., Liu, B., Xie, Z.-t., Wu, S.-t., Yao, Y.-f., Yang, Y.-f., 2020. Network Pharmacology-based Research of Active Components of Albiziae Flos and Mechanisms of Its Antidepressant Effect. Curr Med Sci. 40, 123-129.

Supplement Figure S1: (**A-B**) Protein levels of α-SMA and Vimentin in renal tubules of diabetic mice were detected by immunohistochemistry. ** P < 0.01 *vs*. Ctrl. ## P < 0.01 and # P < 0.05 *vs*. diabetes. (**C**) The migration ability of NRK-52E cells under the HG conditions at various times were detecetd by cell scrath test. ** P < 0.01 and * P < 0.05 *vs*. 0 h. (**D**) The effect of GPS on NRK-52E cells viability was detected by MTT assay. * P < 0.05 *vs*. Ctrl. (**E**) The migration ability of NRK-52E cells after GPS intervention were detected by cell scrath test. ** P < 0.01 *vs*. Ctrl, ## P < 0.01 *vs*. HG. (**F**) The distributions p65 in nucleus and cytoplasm under the HG conditions at various times were detected by western blot. * P < 0.05 *vs*. 0 h. Data was expressed as mean ± SD.

Supplement Figure S2: (**A**) AT1R and GFP expressions were detecetd by western blot after pcDNA3.1-GFP-AT1R plasmid transient transfection. * P < 0.05 *vs*. Ctrl. # P< 0.05 *vs*. HG. ^^ P< 0.01 *vs*. HG with GPS. (**B**) The effect of GPS on migration ability after AT1R over-expression was assessed by cell scrath test. ** P < 0.01 *vs*. Ctrl. ## P< 0.01 *vs*. HG. ^^ P< 0.01 *vs*. HG with GPS. (**C**) The effect of GPS on p65 distribution after AT1R over-expression was assessed by immunofluorescence. Scale bar: 20 μm. (**D**) The distributions of CK2α and IκBα in NRK-52E cells were assessed by immunofluorescence. Scale bar: 20 μm. Data was expressed as mean ± SD.

Supplement Figure S1

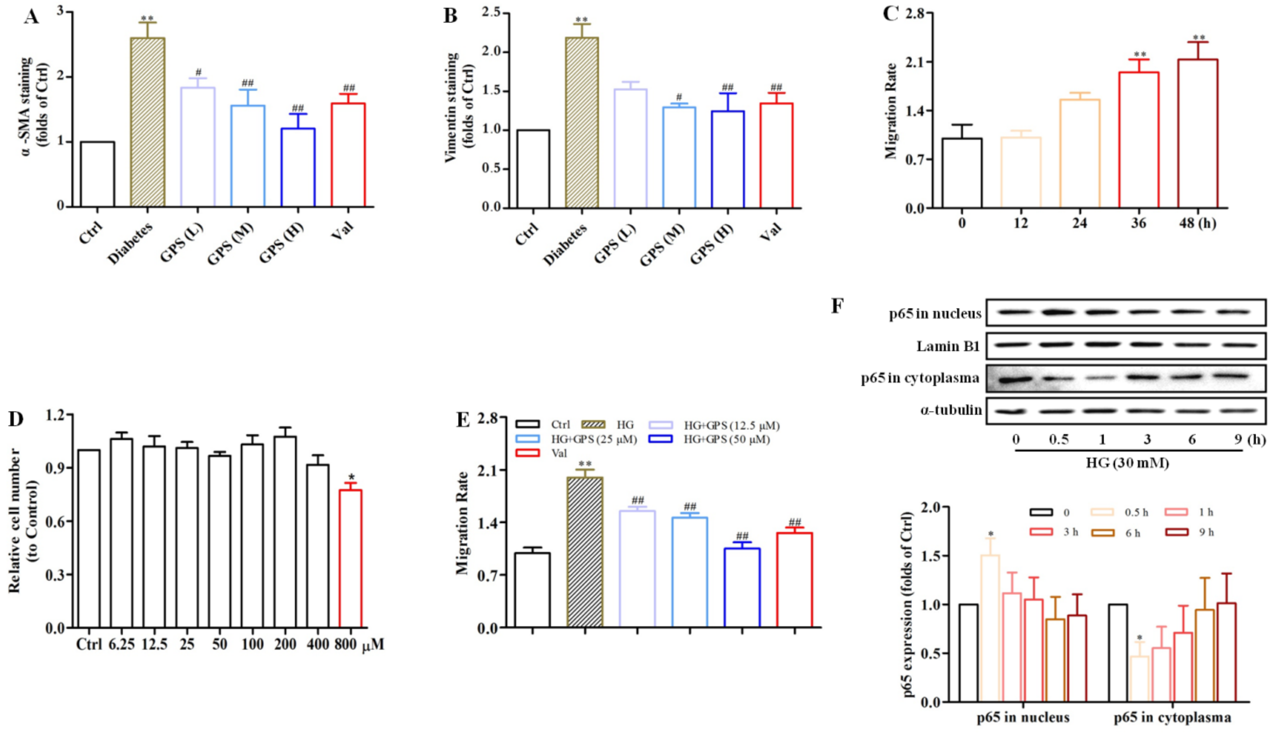

Supplement Figure S2

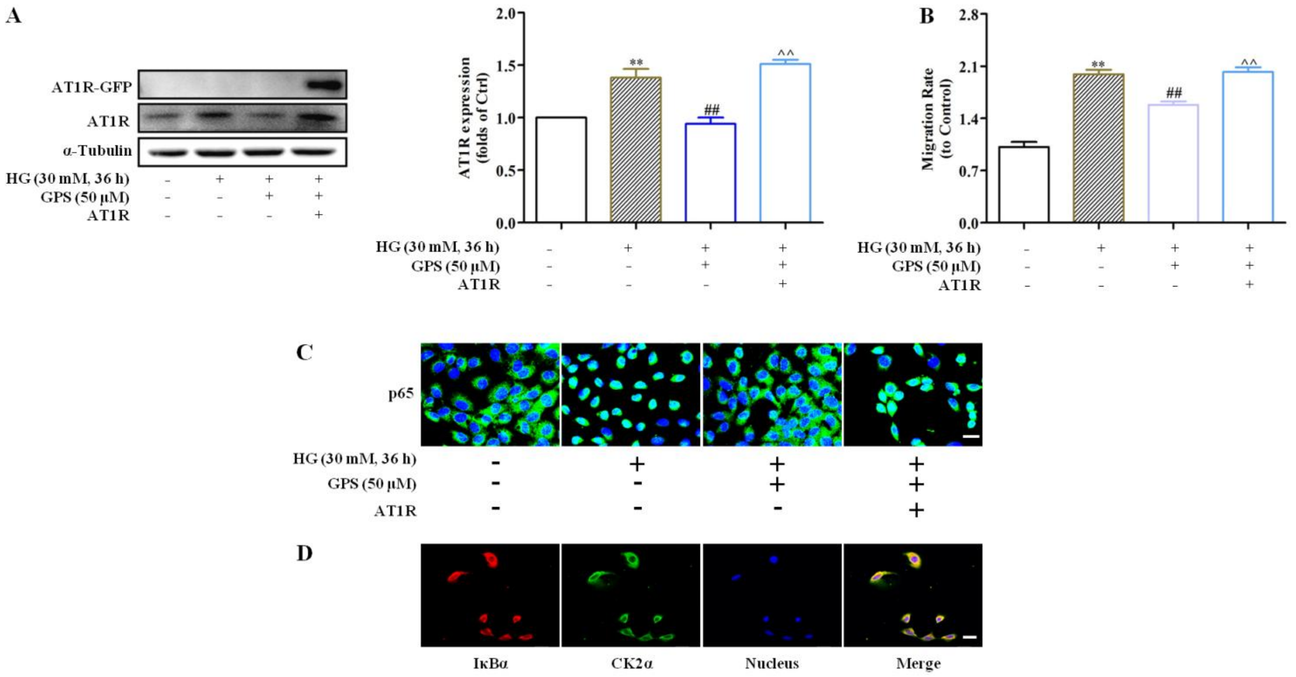

Table legend

Supplement Table 1 KEGG pathway analysis of interacting target (Top 10)

pathway	description	count in network	strength	false discovery rate
Hsa00910	Nitrogen metabolism	3 0f 17	1.87	0.00014
Hsa05219	Bladder cancer	5 0f 41	1.71	7.64e-06
Hsa04215	Apoptosis-multiple species	3 0f 31	1.61	0.00043
Hsa04973	Carbohydrate digestion and absorption	4 0f 42	1.6	5.18e-05
Hsa04964	Proximal tubule bicarbonate reclamation	2 0f 23	1.56	0.0040
**Hsa04614**	**Renin-angiotensin system**	**2 0f 23**	**1.56**	**0.0040**
Hsa05120	Epithelial cell signaling in helicobacter pylori infection	5 0f 66	1.5	1.73e-05
Hsa05212	Pancreatic cancer	5 0f 74	1.45	1.97e-05
Hsa04930	Type 2 diabetes mellitus	3 0f 46	1.43	0.00094
Hsa00052	Galactose metabolism	2 0f 31	1.43	0.0064
